# Relation between Guillain-Barré syndrome and Covid-19: Case-Series

**DOI:** 10.25122/jml-2023-0275

**Published:** 2023-09

**Authors:** Merey Bakytzhanovna Jumagaliyeva, Dinmukhamed Nurniyazovich Ayaganov, Ibrahim Anwar Abdelazim, Samat Sagatovich Saparbayev, Nodira Miratalievna Tuychibaeva, Yergen Jumashevich Kurmambayev

**Affiliations:** 1 Department of Neurology, Psychiatry and Narcology, West Kazakhstan Marat Ospanov Medical University, Aktobe, Kazakhstan; 2 Department of Obstetrics and Gynecology, Faculty of Medicine Ain Shams University, Cairo, Egypt; 3 Department of Normal Physiology, West Kazakhstan Marat Ospanov Medical University, Aktobe, Kazakhstan; 4 Department of Neurology, Psychology and Psychotherapy, Tashkent Medical Academy, Tashkent, Uzbekistan

**Keywords:** Covid-19, GBS, Guillain-Barré syndrome, Covid-19: Corona-virus disease-19, CSF: Cerebro-spinal fluid, CT: Computerized tomography, GBS: Guillain-Barré syndrome, ICU: Intensive care unit, MFS: Miller-Fisher syndrome, MRC: Medical Research Council, PCR: Polymerase chain reaction, SARS: Severe acute respiratory syndrome, TR: Tendon reflexes, UTI: Urinary tract infection, WKMU: West Kazakhstan Medical University

## Abstract

Approximately two-thirds of the Guillain-Barré syndrome (GBS) cases are preceded by upper respiratory tract infection or enteritis. There has been previous documentation of a clear association between Covid-19 and GBS. Covid-19 can affect the nervous tissue either through direct damage or through triggering a host immune response with subsequent development of autoimmune diseases such as GBS. Covid-19 can affect the host`s immune system through the activation and interaction of the T-and B-lymphocytes with subsequent production of antibodies that cross-react with the gangliosides. Depending on the nature of the neuronal autoimmune destruction, the affected individual may have either a demyelinating or axonal subtype of GBS. These subtypes differ not only in symptoms but also in the likelihood of recovery. This report presents two cases of GBS that developed after the respiratory symptoms of Covid-19. Their neurological features indicated demyelination, axonal damage, irritation of spinal nerve roots, and impaired sensory and motor transmission with additional facial nerve palsy in the second-studied case. This case report highlights the relationship between GBS and Covid-19 infection.

## INTRODUCTION

Guillain-Barre syndrome (GBS) is an acute or subacute, rapidly progressing autoimmune disease of the peripheral nervous system (monophasic immune-mediated neuropathy) manifested by limb paraesthesia, muscle weakness, and/or flaccid paralysis [1]. It may also present as defective facial, oculomotor, and bulbar nerves, known as the Miller-Fisher syndrome (MFS) [1, 2]. About two-thirds of the GBS cases present with nerve root pain before the onset of muscle weakness [3]. In most cases of GBS, there are decreased tendon reflexes (TR) in the affected limbs. However, the tendon reflexes may be unchanged in the early stages of the disease [4]. The incidence of GBS is 1.2-2.3 cases per 100,000 individuals [5]. In contrast, for people over the age of 50 years, it can reach 3.3 per 100,000 individuals [6]. For this population, GBS can lead to residual functional disability [7]. Recent reports discovered that severe acute respiratory syndrome (SARS)-Cov-2 (Covid-19) was associated with neurological complications including febrile seizures, headache, dizziness, myalgia, encephalopathy, stroke, and peripheral nerve diseases [8]. About two-thirds of the GBS cases are preceded by upper respiratory infection or enteritis [9]. Covid-19 can affect the nervous tissue either through direct damage or through triggering the host`s immune response [10, 11] with subsequent development of autoimmune diseases such as GBS [12]. This report highlights the relationship between GBS and the COVID-19 infection.

## CASE PRESENTATION

### Case-1

A 23-year-old woman, primipara, pregnant at 30 weeks gestation, was admitted to the infectious diseases hospital of West Kazakhstan Medical University (WKMU) with acute weakness of the lower limbs, nasal voice, cough, shortness of breath, dizziness, and headache. She gave a history of hospital admission, due to a urinary tract infection (UTI) with pregnancy, treated with intravenous antibiotics 3 weeks before the current admission. On admission, the patient presented with a temperature of 37.5°C, blood pressure of 110/65 mmHg, pulse rate of 82 beats/min., and respiratory rate of 22/min, as well as with a positive nasopharyngeal swab polymerase chain reaction (PCR) test for Covid-19. The blood panel showed leukocytosis, lymphocytopenia, and increased lactate dehydrogenase (LDH), while the chest computerized tomography (CT) showed bilateral lower lobe pneumonia. The neurological examination showed decreased right pharyngeal reflex, lower limbs flaccid symmetrical paraparesis [Medical Research Council (MRC) scale 4/5 proximal, and 2/5 distal], lost lower limb reflexes (knee and ankle reflexes), legs tingling, and no signs of meningeal irritation. The electroneurography showed acute motor demyelinating neuropathy (delay in the distal nerve latency, decreased conduction velocity, and decreased amplitude of the action potential). The patient clinically improved after receiving combined antimicrobial medication (azithromycin 500 mg/once daily for 5 days and amoxicillin/clavulanic acid 1 gm/twice daily for 7 days) and intravenous immunoglobulins 0.4 g/kg/day for 5 days (after obstetrics consultation).

### Case-2

A 59-year-old woman was admitted to the intensive care unit (ICU) of WKMU with general weakness associated with chest pain, coughing, and shortness of breath. On admission, she presented with a temperature of 38.5°C, blood pressure of 140/80 mmHg, pulse rate of 86 beats/min., and a respiratory rate was 25/min. The patient had a positive nasopharyngeal swab PCR test for COVID-19, bilateral lower lobe pneumonia, and interstitial pneumonitis (ground glass opacities) on chest CT. The blood panel showed lymphopenia with increased C-reactive proteins and increased LDH. The neurological examination showed bilateral facial paresis, bilateral ptosis, dysphonia, dysphagia, and quadriplegia [Medical Research Council (MRC) scale 1/5 proximal and 0/5 distal in the lower limbs], diffuse muscle hypotonia, tingling with absent hand and feet reflexes with positive tension symptoms of the nerve roots, and positive right Babinski sign. The electroneurography showed acute demyelinating polyneuropathy (sensory and motor with delay in the distal nerve latency, decreased conduction velocity, and decreased amplitude of the action potential). The brain magnetic resonance imaging (MRI) was completely normal and the cerebrospinal fluid (CSF) examination following the lumbar puncture showed an increased immunoglobulin (IgG)/albumin ratio. The COVID-19 PCR test was negative. The patient was diagnosed with classic Miller-Fisher syndrome with cerebrospinal fluid protein-cell dissociation and received intravenous immunoglobulins in a dose of 0.4 g/kg/day for 5 days. Despite all treatment efforts, she developed severe respiratory distress which progressed to respiratory failure, and died. The diagnosis of the COVID-19 infection in the studied cases was based on the nasopharyngeal swab PCR test, blood picture (lymphopenia, increased inflammatory markers such as C-reactive proteins and LDH), and chest CT. The GBS was diagnosed using the World Health Organisation (WHO) international criteria [13] and Brighton Collaboration diagnostic criteria [14].

## Discussion

Roughly two-thirds of the GBS cases are preceded by an upper respiratory infection or enteritis [9]. Gigli *et al* [15], reported an increased incidence of GBS (5.41 times) in 2019-2020. A definite association between Covid-19 and GBS was present in many reports [16-18]. GBS-related polyneuropathy has been reported to occur at the onset Covid-19 symptoms as well as before or after the appearance of Covid-19 symptoms [19-22]. Sedaghat *et al*. [23] reported instances of GBS occurring after the onset of Covid-19 symptoms, while Ottaviani *et al*. [24] reported GBS after the Covid-19 symptoms. Alberti *et al*. [25] reported GBS occurring before the onset of Covid-19 respiratory symptoms in a 71-year-old male. Additionally, Zhao *et al*. [26], presented cases of acute progressive weakness in both legs 8 days before the Covid-19 symptoms. Covid-19 can affect the nervous tissue through direct damage or triggering the host`s immune response [10, 11] with subsequent development of autoimmune diseases such as GBS [12]. Moreover, GBS can occur after vaccination, pregnancy, or a surgical procedure [27]. The SARS-CoV-2 infection may trigger the host`s immune response through the activation and interaction of the T-and B-lymphocytes with subsequent production of antibodies against SARS-CoV-2 [28]. These antibodies cross-react with the gangliosides (sialic acid-containing glycosphingolipids, located on the neuronal cells surface) leading to either autoimmune destruction of myelin sheaths or axons [29-30]. Fantini *et al*. [31] reported a cross-reaction between the SARS-CoV-2 spike saccharides and myelin sheaths or axon gangliosides. A review suggests that SARS-CoV-2 can directly destroy the gangliosides [32]. Depending on the level of neuronal autoimmune destruction, the affected individual may have either a demyelinating or axonal GBS subtype which can differ in symptoms and the probability of recovery [33]. Moreover, some viruses, such as the cytomegalovirus and the chickenpox virus, can cause peripheral neuropathy through direct nerve damage [34]. This report presents two cases of GBS developed after the respiratory symptoms of Covid-19 and their neurological features indicated demyelination, axon damage, irritation of spinal nerve roots, and impaired sensory and motor transmission with additional facial nerve palsy in the second case studied, similar to findings reported by literature. Myckatyn *et al*. [35] also reported facial nerve palsy in 27-50% of the GBS cases.

## CONCLUSION

SARS-CoV-2 is a newly diagnosed virus that affects the nervous system through a para-infectious phenomenon. It is important to consider Covid-19 screening in patients with GBS, as the disease can develop concurrently with the onset of COVID-19 symptoms, after the appearance of the symptoms, or even in the lack of symptoms. Further research is required to confirm the increased incidence of GBS during the Covid-19 pandemic and the definite relation between GBS and Covid-19.

## Data Availability

Further data are available from the corresponding author upon reasonable request.
